# Migratory polyarthritis as a paraneoplastic syndrome in a patient with diffuse large B cell lymphoma: a case report

**DOI:** 10.1186/s13256-018-1700-5

**Published:** 2018-06-26

**Authors:** Kavinga Kalhari Kobawaka Gamage, Mohomed Ismail Mohomed Rifath, Harshini Fernando

**Affiliations:** National Hospital of Sri Lanka, Colombo, Sri Lanka

**Keywords:** Diffuse large B cell lymphoma, Paraneoplastic arthritis, Carcinomatous polyarthritis, Migratory polyarthritis

## Abstract

**Background:**

Diffuse large B cell lymphoma is the commonest histological subtype of non-Hodgkin lymphoma and typically presents as a rapidly enlarging lymph node mass and B symptoms. It is unusual for diffuse large B cell lymphoma to present as carcinomatous polyarthritis which is a type of paraneoplastic arthritis.

**Case presentation:**

We present a case of a 45-year-old Asian man with diffuse large B cell lymphoma presenting with generalized lymphadenopathy, transient macular rash, and migratory polyarthritis involving both upper and lower limb small and large joints. Treatment of the lymphoma but not the routine anti-inflammatory agents resulted in complete resolution of the arthritis suggesting the paraneoplastic nature.

**Conclusions:**

Poor response to routine therapy for inflammatory arthritis should lead to early suspicion of paraneoplastic arthritis which will prompt investigation for an underlying malignancy. Suspicion of carcinomatous polyarthritis should be made in those with migratory polyarthritis and should be thoroughly investigated to exclude underlying malignancy.

## Background

Diffuse large B cell lymphoma (DLBCL) is the commonest histologic subtype of non-Hodgkin lymphoma in almost all parts of the world. It accounts for 25–31% [1] of all cases of non-Hodgkin lymphoma in developed countries [2]. It has a male predominance [3] and the prognosis is generally poor; the poor prognosis is probably related to its aggressive nature compared to other types of lymphoma. The International Prognostic Index has been used over decades to identify patients with poor prognosis.

DLBCL commonly presents as a rapidly growing lymph node mass and in 40% of patients there is primary extranodal and extramedullary involvement [4]. Extranodal involvement can occur virtually in any organ, the stomach being the commonest site. Of all patients with DLBCL, 30% experience systemic B symptoms (fever, weight loss, drenching night sweats) [1]. It is uncommon for patients with DLBCL to present with paraneoplastic arthritis even though T cell lymphomas are known to do so. Here we present a case report of a patient presenting with generalized lymphadenopathy and a transient rash followed by inflammatory type symmetrical polyarthritis who was diagnosed later as having DLBCL. To the best of our knowledge this is the only case report of DLBCL associated with polyarthritis.

## Case presentation

A 45-year-old previously healthy Asian man presented with a history of intermittent fever with chills and rigors over 2 months’ duration. There were associated night sweats, loss of appetite, and loss of weight. There was a history of transient macular rash at the onset of the fever which spontaneously resolved without treatment. Generalized lymphadenopathy was noted by our patient mainly involving cervical, axillary, and inguinal regions over 1 month which became extremely painful a few days prior to his presentation. He had synovitis involving lower limb small joints following the presentation, progressing to lower limb large joints and ultimately upper limb small and large joints over 3 days. He did not have past history or family history of arthritis and he had an unremarkable past medical history. He worked as a mason but had never been exposed to toxic environmental conditions to his knowledge and there was no promiscuous sexual behavior. He did not consume alcohol and he did not smoke tobacco.

On examination at the initial presentation he was emaciated, febrile, and pale. There were bilateral, firm, matted lymph nodes of varying sizes of 2–3 cm in the cervical, axillary, and inguinal regions which were tender. There was tender hepatosplenomegaly. The rest of the examination was normal. However, a few days following admission there was bilateral symmetrical polyarthritis involving both small and large joints of upper and lower limbs with lower limb predominance. There was marked synovitis of distal and proximal interphalangeal joints of lower limbs compared to the rest of his joints.

Laboratory investigations revealed high white cell counts with normocytic anemia. Platelets were within the normal range. His inflammatory markers were high and they were in a rising trend following the onset of arthritis. His liver and renal functions were normal. Rheumatoid factor, anti-cyclic citrullinated peptide, anti-nuclear antibodies, human immunodeficiency virus (HIV) 1 and 2 antibodies, Epstein–Barr virus immunoglubulin G (IgG) and immunoglubulin M (IgM), cytomegalovirus IgG and IgM, and *Toxoplasma* antibodies were all negative or within normal limits. His serum uric acid was marginally elevated. An X-ray of his hands and feet showed soft tissue swelling without evidence of erosions or osteopenia.

His first lymph node biopsy showed a reactive lymph node. The biopsy was repeated due to a strong suspicion of lymphoma. The second lymph node biopsy with immunohistochemistry showed large pleomorphic cells with CD20 positivity and small lymphoid cells with CD3 positivity. The population of T cells was high, but the presence of B cells arranged in cohesive clusters and sheets favored a high grade DLBCL. Contrast-enhanced computed tomography (CT) of his neck, chest, and abdomen staged him at Ann Arbor stage IV.

He was started on non-steroidal anti-inflammatory agents and colchicine for the arthritis but there was no response to treatment. Treatment with rituximab, cyclophosphamide, vincristine, doxorubicin, and prednisolone (RCHOP) commenced; following the first cycle of therapy, his arthritis showed marked response with complete resolution of the synovitis. He completed his chemotherapy but intermittently succumbed to infections. His synovitis completely resolved following chemotherapy.

## Discussion

The differential diagnosis of migratory polyarthritis is broad and includes infectious causes, crystal-induced arthropathy, rheumatoid arthritis, vasculitis syndromes, connective tissue disorders, and spondyloarthropathies [5]. Less common etiologies include metastatic disease and paraneoplastic syndromes such as carcinomatous polyarthritis.

Paraneoplastic rheumatic disorders are defined as rheumatic symptoms resulting from an underlying malignant disease, which is not directly related to a tumor or metastasis. These occur as a result of a wide variety of tumor-derived biologic mediators, such as hormones, peptides, antibodies, cytotoxic lymphocytes, and autocrine and paracrine mediators [6]. Symptoms of paraneoplastic arthritis may occur along with the malignant process or may precede the process. These rheumatic conditions do not respond to conventional treatment and will improve with treatment of the underlying malignancy. Despite being a rare incident, early suspicion of paraneoplastic arthritis encourages investigation for the associated malignancy and early diagnosis.

Carcinomatous polyarthritis, a subtype of paraneoplastic arthritis, has been reported with a variety of solid tumors including lung, colon, breast, ovarian, laryngeal, and pancreatic carcinoma [7–10]. The exact prevalence is unknown but it is thought to be rare. Historically, carcinomatous polyarthritis has been characterized by late age of onset, acute onset, asymmetric joint involvement, a predilection for lower extremity joints, sparing of the wrists and hands, benign radiographic changes, and an absent rheumatoid factor [11].

The history and physical examination can often distinguish carcinomatous polyarthritis from other common causes of polyarticular arthritis. However, differentiating from rheumatoid arthritis can be quite challenging. This distinction is critical for prompt therapy of malignancy. Often, carcinomatous polyarthritis does not fit the presentation historically described. There are no definitive diagnostic tests and, ultimately, clinical suspicion is the most important factor in accurate diagnosis. It should be suspected in patients with new onset of acute migratory arthritis at a relatively late age. The presence of rheumatoid factor and involvement of the wrists and hands should not lessen clinical suspicion, particularly in patients with risk factors for cancer. In fact, DLBCL is the commonest type of lymphoma associated with rheumatoid arthritis [12, 13]. Other chronic inflammatory conditions have also been associated with increased risk of malignant lymphomas [12]. But the association with paraneoplastic arthritis and DLBCL is not well known.

The pointers toward carcinomatous polyarthritis in our case were the typical clinical symptoms and signs with absolute resolution immediately after chemotherapy. With the pattern of the joint involvement carcinomatous polyarthritis was suspected and repeated biopsy of lymph node was done which demonstrated the presence of DLBCL. Therefore we suggest repeated thorough investigations to exclude the presence of an underlying malignancy of patients presenting with migratory type polyarthritis. Also there was poor response to routine anti-inflammatory agents for the arthritis which further suggested the possibility of underlying malignant cause. We could not find any case of DLBCL presenting with carcinomatous polyarthritis and we think this is the first case of carcinomatous polyarthritis associated with DLBCL.

## Conclusions

Poor response to routine anti-inflammatory treatment should suggest the presence of paraneoplastic arthritis. In the presence of migratory polyarthritis with lower limb predominance underlying malignancy should be thoroughly investigated and excluded.

## Abbreviations

DLBCL: Diffuse large B cell lymphoma
